# DBO-AWOA: An Adaptive Whale Optimization Algorithm for Global Optimization and UAV 3D Path Planning

**DOI:** 10.3390/s25072336

**Published:** 2025-04-07

**Authors:** Tao Xu, Chaoyue Chen

**Affiliations:** School of Automation, Shenyang Aerospace University, Shenyang 110136, China; chenchaoy1@163.com

**Keywords:** UAV, path planning, DBO-AWOA, chaotic mapping, nonlinear convergence factors, adaptive inertia mechanisms, dung beetle optimizer

## Abstract

The rapid expansion of unmanned aerial vehicle (UAV) applications in complex environments presents significant challenges in 3D path planning, particularly in overcoming the limitations of traditional methods for dynamic obstacle avoidance and computational efficiency. To address these challenges, this study introduces an adaptive whale optimization algorithm (DBO-AWOA), which incorporates chaotic mapping, nonlinear convergence factors, adaptive inertia mechanisms, and dung beetle optimizer-inspired reproductive behaviors. Specifically, the algorithm utilizes ICMIC chaotic mapping to enhance initial population diversity, a cosine-based nonlinear convergence factor to balance exploration and exploitation, and a hybrid strategy inspired by the dung beetle optimizer to mitigate stagnation in local optima. When evaluated on the CEC2017 benchmark suite, DBO-AWOA demonstrates superior convergence precision and robustness, achieving the lowest minimum and average values across 72% of test functions. In 3D path-planning simulations within mountainous environments, DBO-AWOA generates smoother, shorter, and safer trajectories compared to existing variants, with fitness values reduced by 5–25%. Although the algorithm demonstrates slight instability in highly dynamic hybrid functions, its overall performance marks an improvement in global optimization and UAV path planning.

## 1. Introduction

The rapid advancement of the low-altitude economy has driven the widespread deployment of unmanned aerial vehicle (UAV) systems across sectors such as smart logistics [1], precision agriculture [2], and military operations [3]. However, large-scale implementation faces significant technical challenges. According to the 2023 annual report of the International UAV Systems Association, the global commercial UAV market is projected to surpass USD 120 billion by 2025. Yet, limitations in current route-planning technologies hinder approximately 23% of potential applications. In complex environments—including urban canyons, mountainous terrain, and dynamically controlled airspace—conventional two-dimensional path-planning approaches, which rely on geometric constraints, exhibit critical shortcomings. These include inadequate three-dimensional spatial modeling and substantial delays in dynamic obstacle avoidance [4].

Classical path-planning approaches predominantly rely on adaptations of the A* algorithm [5] and the rapidly exploring random tree (RRT) framework [6]. However, these methods encounter exponential increases in computational complexity when applied to high-dimensional continuous state-space planning. A paradigm shift toward intelligent optimization algorithms has provided a promising avenue to address this challenge.

Swarm intelligence techniques, including particle swarm optimization (PSO) [7], genetic algorithms (GAs) [8], and the whale optimization algorithm (WOA) [9], have emerged as key research directions for UAV path planning. Considerable efforts have focused on refining these algorithms or integrating multiple swarm intelligence strategies to improve high-dimensional optimization and route efficiency. Liu et al. [10] introduced a hybrid multi-strategy artificial rabbit optimization (HARO) algorithm for UAV path planning in complex environments. By integrating a dual exploration switching strategy with a population migration memory mechanism, HARO effectively balances exploration and exploitation, enhancing search efficiency and minimizing the likelihood of local optima. Jiang et al. [11] proposed a UAV 3D path generation method combining a partially observable Markov decision process (POMDP) with an improved grey wolf optimizer (GWO), demonstrating enhanced convergence and constraint-handling capabilities. Liu et al. [12] introduced a modified sparrow search algorithm, CASSA, incorporating chaotic mapping and adaptive inertia weighting to improve convergence speed. Comparative simulations indicated superior path-planning performance over conventional sparrow search algorithms. Yu et al. [13] advanced PSO by integrating a simulated annealing (SA) mechanism, strengthening global optima updates and mitigating local convergence issues through dimension-based learning strategies. Wang et al. [14] developed an enhanced Butterfly Algorithm featuring a population reset strategy and hybrid particle swarm optimization, validated through simulation. He et al. [15] proposed the HIPSO-MSOS hybrid algorithm, integrating enhanced PSO with modified symbiotic search, yielding superior multi-objective optimization performance for multi-UAV 3D path planning. Similarly, Pan et al. [16] introduced the dual-learning golden eagle optimizer (GEO-DLS), incorporating personal exemplar learning and mirror reflection mechanisms to improve global search efficiency, successfully addressing UAV path planning in power inspection scenarios.

Reinforcement learning has also demonstrated notable advantages in UAV navigation. Yu et al. [17] developed a reinforcement learning-driven multi-strategy cuckoo search algorithm (RL-MCS), integrating adaptive strategy selection and dynamic parameter tuning to achieve superior optimization performance in both benchmark functions and complex 3D flight environments. Xie et al. [18] employed deep reinforcement learning for 3D path planning, leveraging local information and relative distance instead of global data, achieving greater stability and learning efficiency compared to Deep Q-Network and Deep Recurrent Q-Network models. Alpdemir [19] introduced a reinforcement learning framework tailored for tactical UAV navigation under dynamic radar threats, incorporating probabilistic engagement modeling and geometry-aware maneuver optimization to address sparse reward challenges in contested electromagnetic environments. Lin et al. [20] proposed a fixed-horizon constrained reinforcement learning framework for decision-making and planning tasks. Experimental results demonstrate that the framework outperforms rule-based, imitation learning-based, and traditional reinforcement learning approaches, underscoring its effectiveness in handling complex planning scenarios.

Swarm intelligence algorithms have gained significant attention in global optimization, harnessing collective dynamics to address complex optimization challenges. In UAV path planning, these algorithms typically encode population individuals as a sequence of vector coordinates, representing both position and attitude to define the complete flight trajectory. However, as the number of path nodes increases, the dimensionality of the problem grows exponentially, leading to heightened computational complexity and making the pursuit of a globally stable optimal solution increasingly difficult. To address this challenge, the whale optimization algorithm (WOA) [9] has emerged for its robust performance across low- to high-dimensional optimization tasks. Inspired by the hunting strategies of whales, including circling behavior and bubble-net foraging, WOA is computationally efficient, easy to implement, and has found widespread application across various fields. Despite its strengths, the WOA faces several limitations, such as susceptibility to local optima, slow convergence, and limited global search capacity. Overcoming these challenges is vital for improving WOA’s effectiveness in real-world applications, such as UAV path planning.

To overcome these limitations, researchers have proposed various enhancements to improve the performance of the WOA. Kaur et al. [21] explored the incorporation of various chaotic mappings into the WOA, analyzing their impact on optimization performance. Qu et al. [22] proposed the Spiral-Enhanced Whale Optimization Algorithm (SEWOA), introducing a nonlinear time-varying self-adaptive perturbation strategy alongside an Archimedean spiral structure, with experimental validation confirming its effectiveness. Sun et al. [23] further refined the WOA by integrating a tent map and novel iteration-based update mechanisms for the convergence factor, inertia weight, and optimal feedback strategy, demonstrating superior performance in benchmark function tests. Similarly, Fan et al. [24] enhanced the WOA with a tent map, adaptive inertia weighting, and an opposition-based learning mechanism, yielding improved adaptability to high-dimensional global optimization problems, as verified through numerical experiments.

Building on previous advancements, this study proposes an adaptive whale optimization algorithm enhanced with dung beetle optimization (DBO-AWOA). Compared to the standard WOA, DBO-AWOA improves the algorithm’s ability to escape local optima while enhancing convergence accuracy, making it particularly well suited for UAV path planning. The key enhancements introduced in DBO-AWOA include the following:

(1) The ICMIC chaotic mapping is employed to optimize initial population distributions, improving solution quality.
(2) A nonlinear convergence factor is introduced to dynamically balance exploration and exploitation.
(3) An adaptive inertia strategy is integrated into the spiral position updating mechanism, facilitating escape from local optima.
(4) Drawing on reproductive behaviors from the dung beetle optimization (DBO) algorithm, a novel optimization mechanism is designed to strengthen local search capability.

The structure of this paper is as follows: Section 2 provides an overview of the WOA and details the update mechanisms introduced in DBO-AWOA. Section 3 evaluates the algorithm’s performance through benchmark tests and simulations. Finally, Section 4 presents the conclusions of the study.

## 2. DBO-AWOA

### 2.1. Whale Optimization Algorithm

The whale optimization algorithm (WOA) [9] is inspired by the distinctive spiral bubble-net hunting strategy of humpback whales. It models optimization by simulating three key predatory behaviors: encircling prey, spiral position updating, and stochastic search. The corresponding mathematical formulation is as follows: In this algorithm, a population of size *n* is considered within a *D*-dimensional solution space. The position of each individual is represented as ${\vec{X}}_{i}=\left(\vec{X_{i}^{1}},\vec{X_{i}^{2}},\vec{X_{i}^{3}},\ldots ,\vec{X_{i}^{D}}\right),i=1,2,3,\ldots N$.

#### 2.1.1. Encircling Prey

Individual whales iteratively move toward the current optimal solution following the update mechanisms defined in Equations (1)–(3):

(1) $$\vec{D}=\left|\vec{C}\cdot \vec{X}*\left(t\right)-\vec{X}\left(t\right)\right|$$

(2) $$\vec{X}\left(t+1\right)=\vec{X}*\left(t\right)-\vec{A}\cdot \vec{D}$$

(3) $$\vec{A}=2\cdot \vec{a}\cdot {\vec{r}}_{1}-\vec{a}$$

The convergence factor, denoted as $\vec{a}$, is defined as $\vec{a}=2-\frac{2t}{T}$, where *T* represents the maximum number of iterations and *t* denotes the current iteration count. This parameter dynamically regulates the balance between exploration and exploitation and operates alongside $\vec{X}*\left(t\right)$, which represents the best solution identified at each iteration.

#### 2.1.2. Bubble-Net Attacking Method

The control parameter *p* governs the selection between the shrinking encircling mechanism and the spiral model. Here, *p* is a random number in the interval [0, 1]. When p≥0.5, the whale updates its position along a spiral trajectory:

(4) $$\vec{X}\left(t+1\right)={\vec{D}}^{\prime }\cdot e^{bl}\cdot \cos\left(2\pi l\right)+\vec{X}*\left(t\right)$$

(5) $${\vec{D}}^{\prime }=\left|\vec{X}*\left(t\right)-\vec{X}\left(t\right)\right|$$

The parameter *b* controls the spiral shape constant, while *l* is a random number uniformly distributed in the interval [−1,1].

#### 2.1.3. Random Search Phase

When $|\vec{A}|>1$, the individual randomly selects a reference target:

(6) $$\vec{X}\left(t+1\right)={\vec{X}}_{rand}-\vec{A}\cdot {\vec{D}}_{rand}$$

(7) $${\vec{D}}_{rand}=\left|\vec{C}\cdot {\vec{X}}_{rand}-\vec{X}\right|$$

Here, $\vec{X_{rand}}$ denotes the position of a randomly selected individual within the population.

### 2.2. DBO-AWOA

#### 2.2.1. ICMIC Chaotic Mapping

The ICMIC chaotic map is a highly complex dynamical system characterized by strong nonlinear behavior. Its Lyapunov exponent is significantly larger than that of traditional logistic chaotic mapping, indicating greater sensitivity to initial conditions. The mathematical formulation of the ICMIC mapping is given by

(8) $$x_{k+1}=\sin\left(\alpha /x_{k}\right)$$

**Figure 1:**
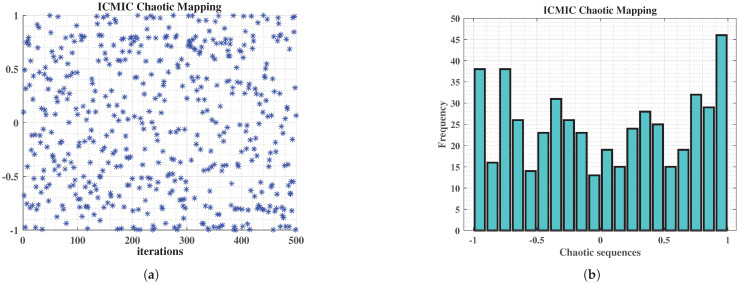
ICMIC chaotic mapping. (**a**) Scatter plot; (**b**) probability histogram.

Here, $x_{k}$ represents the value at the *k*-th iteration, and α is the control parameter. When α>0.6, the system exhibits typical chaotic behavior. To illustrate this, a scatter plot of 500 points is presented, depicting the distribution of generated values on a two-dimensional plane (Figure 1, left). Additionally, the probability histogram of the generated values is shown on the right. In this study, α is set to 0.6.

#### 2.2.2. Nonlinear Convergence Factor

In the WOA, the convergence coefficient $\vec{a}$ plays a crucial role in determining the algorithm’s search efficiency. In the conventional WOA framework, $\vec{a}$ follows a linear decay from 2 to 0. While this approach facilitates strong global exploration in the initial stages, it often results in slow convergence. In later iterations, although the convergence rate increases, the algorithm is prone to becoming trapped in local optima, particularly when solving multimodal optimization problems.

**Figure 2:**
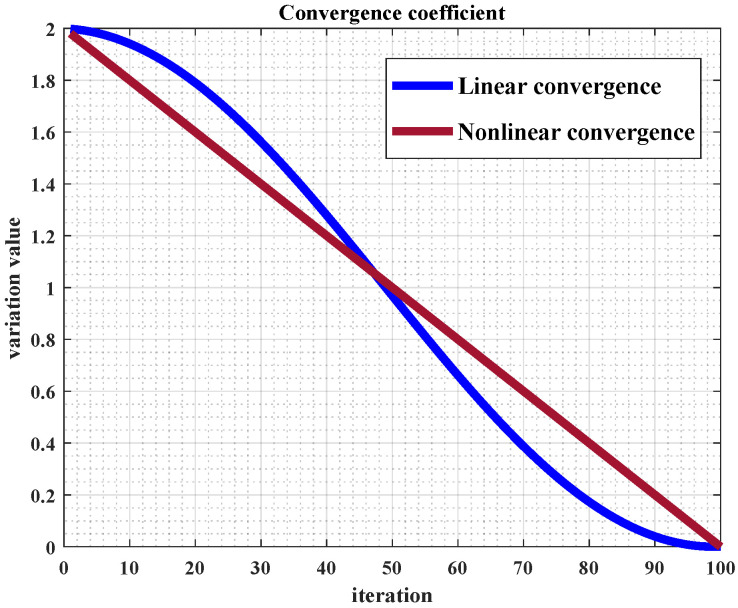
Convergence coefficient.

To overcome these limitations, we propose a nonlinear decay strategy for $\vec{a}$, as illustrated in Equation (9), which replaces the conventional linear adjustment. This modification enhances the WOA’s performance by maintaining effective global exploration in the early stages while ensuring stable convergence and improved local optima avoidance in later phases. By achieving a more balanced trade-off between exploration and exploitation, the proposed strategy enhances robustness in complex optimization scenarios. The variation of the value $\vec{a}$ is depicted in Figure 2.

(9) $$a=1+\cos(\pi \cdot \frac{t+1}{T})$$

#### 2.2.3. Adaptive Inertia Mechanism

Balancing global exploration and local exploitation remains a fundamental challenge in metaheuristic optimization. Traditional algorithms, such as particle swarm optimization (PSO) and the whale optimization algorithm (WOA), typically rely on fixed or linearly decaying parameters to regulate this trade-off. However, such static strategies often struggle to adapt to dynamic search landscapes, particularly in high-dimensional or multimodal optimization problems.

To overcome this limitation, we introduce an adaptive inertia weight mechanism that dynamically adjusts search behavior in response to real-time feedback on population diversity and convergence status. This adaptive approach enhances the algorithm’s ability to navigate complex search spaces, improving both convergence efficiency and solution quality.

(10) $$w=|cos(\pi \cdot \frac{t}{T})|$$

Therefore, Equation (4) becomes

(11) $$\vec{X}\left(t+1\right)={\vec{D}}^{\prime }\cdot e^{bl}\cdot \cos\left(2\pi l\right)+\vec{X}*\left(t\right)\cdot w$$

Furthermore, this study introduces an enhancement to *l*, ensuring that it is not merely a pseudo-random number. The modified formulation is given by

(12) $$a_{2}=-1-\frac{t}{T}$$

(13) $$l=rand\cdot a_{2}+1$$

#### 2.2.4. Fusion with Dung Beetle Algorithm

The dung beetle algorithm [25] is a swarm intelligence optimization technique inspired by the ecological behaviors of dung beetles in natural ecosystems. This method models four key species-specific behaviors—ball-rolling, reproduction, foraging, and stealing—to establish position-updating mechanisms, enabling efficient solutions to complex optimization problems.

In the original whale optimization algorithm (WOA), when $|\vec{A}|\;>1$, the algorithm simulates the encircling prey behavior by updating whale positions based on the global best solution. However, this approach exclusively relies on the global optimum to guide population movements, overlooking the exploitation of individual historical search experiences. As a result, the algorithm exhibits limited local search capability, often leading to premature convergence in complex optimization landscapes.

To address this limitation, we integrate the reproductive behavior mechanism of the dung beetle algorithm into the WOA framework. This hybrid approach allows whales to incorporate both the best solution and their own historical search information during position updates. By balancing global exploration with enhanced local exploitation, the modified algorithm mitigates stagnation in local optima while preserving robust convergence efficiency.

The reproductive behavior of female dung beetles involves burying dung balls at species-specific soil depths for spawning. In the algorithm, this mechanism is modeled by introducing safety zone boundaries, which impose spatial constraints to mitigate excessive population clustering and enhance search diversity.

(14) $$Lb^{*}=\max\left(\vec{X}*\left(t\right)\cdot \left(1-R\right),Lb\right)$$

(15) $$Ub^{*}=\min\left(\vec{X}*\left(t\right)\cdot \left(1+R\right),Ub\right)$$

Here, Lb and Ub denote the lower and upper bounds of the search space, respectively, while R=1−t/T represents a dynamic factor that decreases over iterations.

The spawning position is determined according to a uniform distribution:

(16) $$\vec{X}\left(t+1\right)=\vec{X}*\left(t\right)+b_{1}\cdot \left(\vec{X}\left(t\right)-Lb^{*}\right)+b_{2}\cdot \left(\vec{X}\left(t\right)-Ub^{*}\right)$$

In this equation, $b_{1}$ and $b_{2}$ denote two independent random vectors of size 1×d, with *d* representing the dimensionality of the optimization problem.

Thus, Equation (2) is updated to Equations (17) and (18) by fusing the adaptive inertia weight:

(17) $$\vec{u}=\vec{X}*\left(t\right)-\vec{A}\cdot \vec{D}$$

(18) $$\vec{X}\left(t+1\right)=\vec{X}*\left(t\right)\cdot w+\left(\vec{u}-Lb\right)\cdot rand+\left(\vec{u}-Ub\right)\cdot rand$$

Here, rand denotes a uniformly distributed random number within the interval [0,1].

#### 2.2.5. The Pseudocode of DBO-AWOA

The pseudocode of the WOA algorithm is demonstrated in Algorithm 1.
**Algorithm 1** DBO-AWOA.  1:Initialize the Whales population ${\vec{X}}_{i}$ by ICMIC chaotic mapping through Equation (26)  2:Calculate the fitness of each search agent  3:$\vec{X}*\left(t\right)$ = the best search agent  4:**for** t=1,2,…,T **do**  5:       **for** i=1,2,…,N **do**  6:       Update $\vec{A}$, $\vec{c}$ and *p*  7:       Update $\vec{a}$ by Equation (9)  8:       Update *w* by Equation (10)  9:       Update *l* by Equation (13)10:       **if** *p* < 0.5 **then**11:        **if** $|\vec{A}|\;\leq 1$ **then**12:        Update ${\vec{D}}^{\prime }$ by the Equation (5)13:        Update the position of the current search agent by the Equations (17) and (18)14:        **else**15:        Update ${\vec{D}}_{rand}$ by the Equation (7)16:        Update the position of the current search agent by the Equation (6)17:        **end if**18:       **else**19:        Update the position of the current search agent by the Equation (11)20:       **end if**21:       **end for**22:       Check if any search agent goes beyond the search space and amend it23:       Calculate the fitness of each search agent24:       Update $\vec{X}*\left(t\right)$ if there is a better solution25:**end for**26:return $\vec{X}*\left(t\right)$

## 3. Experiment and Analysis

### 3.1. CEC2017 Test

The computational experiments were conducted on a Windows 11 64-bit operating system, equipped with an Intel® Core™ i7-10510U processor (1.8 GHz base frequency) and 16 GB of RAM. All simulations were implemented using MATLAB R2024a (MathWorks, Inc., Natick, MA, USA). And the CEC (Conference on Evolutionary Computation) test functions are widely utilized for the evaluation of optimization algorithms due to their standardized and diverse benchmark problems, which include unimodal, multimodal, and hybrid functions. These functions present challenging environments, such as high-dimensional spaces and intricate landscapes, that allow researchers to assess an algorithm’s capacity to balance exploration and exploitation. Given these advantages, we employ it to assess the performance of the algorithm.

#### 3.1.1. Comparison with WOA Variants

To thoroughly assess the convergence ability and robustness of the proposed DBO-AWOA algorithm, extensive experiments were conducted using the CEC2017 benchmark suite [26], which consists of 30 test functions grouped into four categories: unimodal (F1 and F3), simple multimodal (F4–F10), hybrid (F11–F20), and composition functions (F21–F30). The specific functions utilized in this study are summarized in Table 1. A comparative analysis was carried out against six state-of-the-art variants: WOA [9], CWOA1 [21], CWOA2 [21], SEWOA [22], and MSWOA [23], JSWOA [24]. The parameter settings for all algorithms are detailed in Table 2. Performance metrics, including minimum (Min), average (Avg), and standard deviation (Std) values, were calculated over 30 independent runs. The benchmark function evaluation results are presented in Table 3.

DBO-AWOA demonstrated superior performance across most test functions, highlighting its robust global search capabilities.

In the case of unimodal functions, DBO-AWOA exhibited faster convergence rates and improved accuracy relative to traditional WOA variants. The incorporation of ICMIC chaotic mapping enhanced the initial population diversity, while the nonlinear decay of the convergence factor maintained a dynamic balance between exploration and exploitation. This dual approach effectively reduced premature stagnation and ensured steady progress toward the global optimum, particularly in high-dimensional continuous search spaces.

For multimodal functions, DBO-AWOA demonstrated strong global search capabilities, owing to its adaptive inertia weight and dung beetle-inspired reproductive behavior. These mechanisms effectively reduced the likelihood of convergence to local optima. In the case of F8, DBO-AWOA achieved the lowest values for Min, Avg, and Std, outperforming other algorithms. The combination of adaptive inertia weight and dung beetle-inspired reproductive behavior helped prevent premature convergence. However, DBO-AWOA still exhibited some limitations, as seen in F10, where its convergence ability placed it in the mid-range of the algorithm rankings.

For hybrid functions, except for F11, F12, F15, F19, and F20, DBO-AWOA exhibited excellent performance and robustness, demonstrating significant potential for further improvement. For instance, in F12, although DBO-AWOA achieved a competitive Min value of $6.39\times 10^{8}$, its Avg value of $1.71\times 10^{9}$ was 85% lower than that of JSWOA ($1.12\times 10^{10}$). However, the Std value of $2.70\times 10^{8}$ indicated instability in highly dynamic sub-function interactions, highlighting an area where the algorithm could be further optimized.

In the composition functions, DBO-AWOA performed excellently in most scenarios. However, a slight overemphasis on global exploration during later iterations occasionally compromised precision, particularly in composition problems with tightly coupled sub-functions. This highlights the need for context-aware parameter adaptation. For instance, in F28, DBO-AWOA’s Avg value of $4.91\times 10^{3}$ slightly lagged behind WOA’s value of $4.09\times 10^{3}$, likely due to excessive global exploration in the final iterations.

In summary, despite encountering some challenges in certain functions, the overall performance of DBO-AWOA is both reliable and highly effective for addressing optimization problems.

#### 3.1.2. Comparison with Mainstream Optimization Algorithm

In this section, we compare the proposed algorithm with several competitive algorithms, including PSO [7], DBO [25], GJO [27], ETO [28] and SCA [29]. To systematically assess the performance of different algorithms, this study employs selected test functions from the CEC2017 benchmark suite (F3, F5, F7, F12, F13, F22, F26, F28) for comparative analysis. Each benchmark function was executed 30 times, with 500 iterations, a population size of 30, and dimensionality of 30. The detailed results of the comparison are presented in Table 4.

DBO-AWOA exhibited superior performance in most benchmark functions (Table 4). In the unimodal function F3, it achieved a minimum value of $4.31\times 10^{4}$, outperforming ETO $\left(4.68\times 10^{4}\right)$ and SCA $\left(5.08\times 10^{4}\right)$, with low standard deviation and average values, indicating high stability. However, in the hybrid function F12, DBO-AWOA demonstrated a relatively weaker performance, although its low standard deviation suggests consistent reliability. Similarly, for the composition function F22, DBO-AWOA attained a minimum fitness of $3.04\times 10^{3}$, improving by 7.5% over GJO ($3.28\times 10^{3}$) and 47% over PSO $\left(5.77\times 10^{3}\right)$, = highlighting its effectiveness in maintaining a balanced exploration–exploitation trade-off in complex search spaces. Overall, DBO-AWOA delivers strong optimization performance, with the exception of F12.

While minor instability in hybrid functions indicates potential for further refinement—such as context-aware parameter tuning—DBO-AWOA’s overall performance represents a notable advancement in metaheuristic optimization.

#### 3.1.3. Ablation Study of DBO-AWOA

To systematically assess the contributions of the four enhancement strategies incorporated into DBO-AWOA, ablation experiments were carried out using eight benchmark functions of the CEC2017 test set (F3, F5, F7, F12, F13, F22, F26, F29). The analysis focused on four variants of the original WOA algorithm: IWOA (WOA with ICMIC chaotic mapping), NWOA (WOA with the nonlinear convergence factor), AWOA (ETO with adaptive inertia mechanism) and DWOA (WOA with DBO reproductive behavior mechanism). Detailed results are presented in Table 5.

The results (Table 5) highlight the distinct contributions of each enhancement. Notably, DBO-AWOA achieves superior performance across all benchmarks, with the exception of F12 and F13.

Incorporating ICMIC chaotic mapping enhanced initial population diversity, facilitating broader exploration of the search space. For the unimodal function F3, IWOA reduced the average fitness (Avg) by 38% compared to WOA ($3.98\times 10^{5}$ vs. $2.44\times 10^{5}$), underscoring its improved exploratory capability in the early iterations. However, in the hybrid function F13, IWOA alone yielded a higher minimum $7.63\times 10^{5}$ than WOA $2.13\times 10^{6}$, suggesting that, in the absence of supplementary mechanisms, its local exploitation remained constrained.

The cosine-based nonlinear decay of the convergence factor dynamically regulated the balance between exploration and exploitation. For F13, this strategy achieved the highest performance among all variants. In F12, NWOA reduced the average fitness to $4.16\times 10^{8}$, outperforming WOA ($4.92\times 10^{8}$), indicating enhanced capability in mitigating premature convergence within complex hybrid landscapes.

The adaptive inertia weight improved the algorithm’s capacity to escape local optima by dynamically adjusting search intensity. In composition function F22, AWOA achieved a 26% reduction in average fitness ($8.98\times 10^{3}$) compared to the WOA ($8.21\times 10^{3}$), demonstrating enhanced exploitation efficiency in later iterations.

The integration of dung beetle-inspired reproductive behavior enhanced local search efficiency. In F7, DWOA achieved the best average fitness, highlighting its effectiveness in navigating tightly coupled hybrid functions by maintaining spatial constraints and preserving population diversity.

These findings highlight that the superiority of DBO-AWOA stems from the synergistic integration of its key components. Chaotic mapping enhances population diversity, nonlinear convergence factors ensure a dynamic balance between exploration and exploitation, adaptive inertia optimizes local refinement, and dung beetle-inspired mechanisms mitigate stagnation. While each enhancement contributes to incremental gains, their combined effect drives robust optimization across complex and diverse landscapes.

### 3.2. Path-Planning Simulation

This section presents the design of the fitness function and reports on experiments conducted for UAV path planning in mountainous simulation environments.

#### 3.2.1. Fitness Function Design

**Figure 3:**
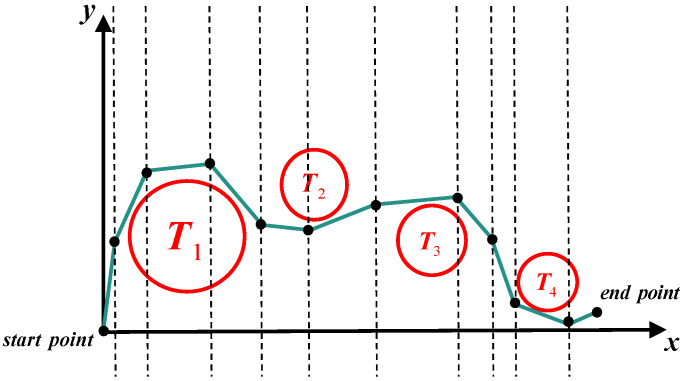
Path representation.

When performing path planning, it is crucial to consider not only the length and algorithm runtime but also additional factors, such as collision avoidance, smoothness, and flight altitude, due to the complexity of the environments encountered. In this study, the UAV’s coordinates in a three-dimensional environment are represented by $\left(x_{i},y_{i},z_{i}\right)$. The path points are first identified through the optimization algorithm, followed by interpolation, ultimately yielding a complete path, as illustrated in Figure 3.

In three-dimensional path planning, flight distance serves as a critical evaluation metric due to its significant influence on mission efficiency and safety. Optimizing flight distance directly impacts energy consumption, as shorter paths reduce the propulsion power required, thereby enhancing the UAV’s operational range and endurance. Furthermore, minimizing flight distance results in shorter mission completion times, improving the UAV’s responsiveness in time-sensitive operations, such as emergency response and package delivery.

(19) $$length=\sum_{i=1}^{m}\sqrt{{\left(x_{i+1}-x_{i}\right)}^{2}+{\left(y_{i+1}-y_{i}\right)}^{2}+{\left(z_{i+1}-z_{i}\right)}^{2}}$$

In the equation, *m* denotes the number of waypoints following UAV path interpolation, and $x_{i+1}$ represents the (i+1)-th waypoint.

**Figure 4:**
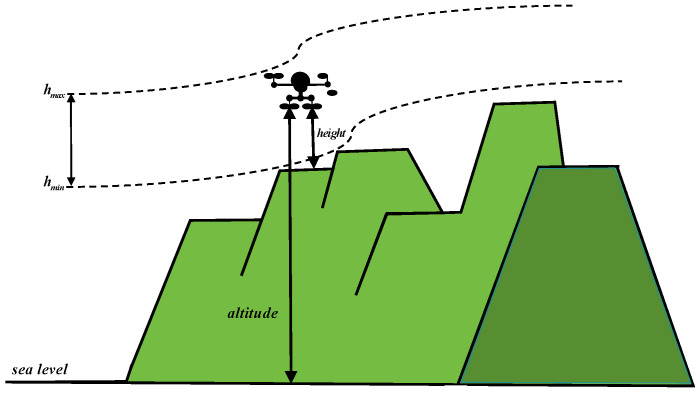
Altitude constraint.

During flight, altitude is a critical factor that influences both mission safety and efficiency. Adjusting flight altitude allows UAVs to avoid obstacles and comply with airspace regulations, particularly in complex environments such as urban areas and mountainous terrains. Optimizing flight altitude reduces the risk of collisions and improves mission success rates. Moreover, altitude directly impacts energy consumption and overall flight efficiency. Higher altitudes may offer more favorable wind conditions and reduced air resistance, potentially lowering energy expenditure. However, excessive altitude increases the power requirements for lift and propulsion. Consequently, optimizing flight altitude requires a balance between energy efficiency and safe navigation, as depicted in Figure 4. The evaluation of flight altitude in this study is represented by Equation (20).

(20) $$altitude\;threat=\sqrt{\frac{1}{m}\sum_{i=1}^{m}\left(max{\left(0,z_{i}-h_{max}\right)}^{2}+max{\left(0,h_{\min}-z_{i}\right)}^{2}\right)}$$

where $h_{min}$ and $h_{max}$ denote the minimum and maximum flight altitudes for path planning, respectively.

**Figure 5:**
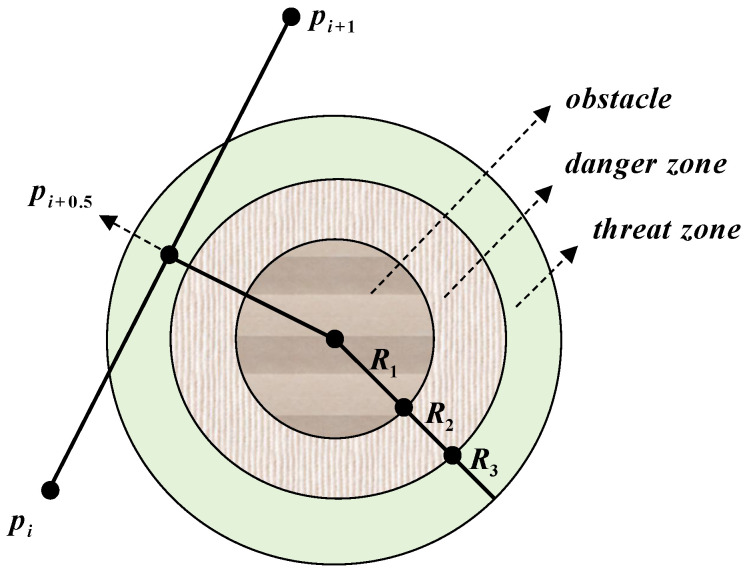
Collision constraint.

Furthermore, the distance to potential collision objects is a critical factor influencing mission safety and success. Ensuring a sufficient distance from obstacles is essential for safe navigation, mitigating collision risks, and improving the overall reliability of UAV operations. This metric becomes particularly crucial in complex environments, where UAVs must maneuver around multiple obstacles while simultaneously optimizing flight paths to achieve mission-specific objectives efficiently, as depicted in Figure 5.

where $p_{i}$ and $p_{i+1}$ denote the *i*-th and (i+1)-th waypoints, respectively. The Euclidean distance $d_{i}$ between the midpoint $p_{i+0.5}$ and the center of the obstacle is used to evaluate the collision threat level. The expression for collision threat is provided in Equation (21).

(21) $$collision\;threat=\left\{\begin{array}{cc} 0 & if\;\;d_{k}>R_{3},\\ d_{k}-rand\cdot R_{2} & if\;\;R_{2}<d_{k}\leq R_{3},\\ d_{k}-R_{1} & if\;\;R_{1}<d_{k}\leq R_{2},\\ \infty & if\;\;d_{k}\leq R_{1}. \end{array}\right.$$

**Figure 6:**
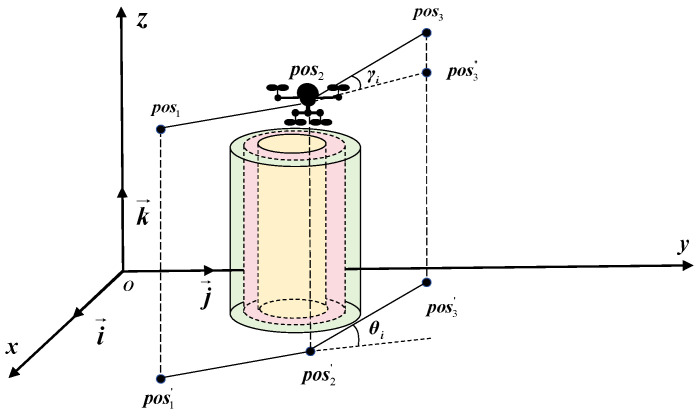
Angle constraint.

Path smoothness is a critical metric for evaluating UAV performance, significantly influencing both operational efficiency and safety. A smooth flight path ensures continuous and differentiable motion, enabling the UAV to navigate without abrupt directional or velocity changes. This reduces the likelihood of instability, lowers energy consumption, and mitigates collision risks. Additionally, smooth paths improve sensor and communication system performance, facilitating high-quality data acquisition and transmission. For UAVs carrying passengers, smoothness enhances ride comfort by minimizing jolts and sudden movements. In summary, path smoothness plays a vital role in optimizing UAV operations in complex environments, ensuring safe, efficient, and reliable mission execution, as illustrated in Figure 6.

In UAV 3D path planning, the horizontal turning angle $\theta_{i}$ and vertical climbing angle $\gamma_{i}$ are key parameters. The waypoints along the UAV’s flight trajectory are represented as ${\mathrm{pos}}_{1}$, ${\mathrm{pos}}_{2}$, and ${\mathrm{pos}}_{3}$, with their corresponding horizontal projections denoted as ${\mathrm{pos}}_{1}^{\prime }$, ${\mathrm{pos}}_{2}^{\prime }$, and ${\mathrm{pos}}_{3}^{\prime }$, respectively.

(22) $$\theta_{i}=\arccos\left(\frac{D_{i-1}\cdot D_{i}}{\left|\right|D_{i-1}\left|\right|\cdot \left|\right|D_{i}\left|\right|}\right)$$

(23) $$D_{i}=\left(x_{i+1}-x_{i},y_{i+1}-y_{i}\right)$$

(24) $$\gamma_{i}=\arctan\left(\frac{z_{i+1}-z_{i}}{\sqrt{{\left(x_{i+1}-x_{i}\right)}^{2}+{\left(y_{i+1}-y_{i}\right)}^{2}}}\right)$$

(25) $$angle\;threat=\sum_{i=2}^{m}w_{\theta }\cdot \theta_{i}+\sum_{i=1}^{m-1}w_{\gamma }\cdot \gamma_{i}$$

Here, $w_{\theta }$ and $w_{\gamma }$ denote the weighting coefficients for $\theta_{i}$ and $\gamma_{i}$.

Considering factors such as path length, flight altitude, collision avoidance, and path smoothness, the overall objective function can be expressed as follows:

(26) $$fitness=w_{1}\cdot length+w_{2}\cdot altitude\;threat+w_{3}\cdot collision\;threat+w_{4}\cdot angle\;threat$$

#### 3.2.2. Simulation Experiment

**Figure 7:**
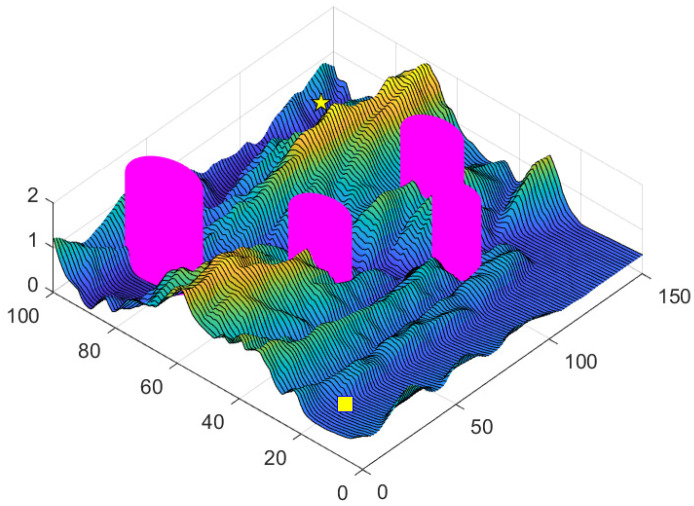
Peak environment.

In this section, a mountain model with dimensions 150×100×2km is employed for path-planning simulations, assuming idealized environmental and weather conditions. Several no-fly zones, including air defense and radar monitoring areas, are designated within the mountainous terrain, as depicted in Figure 7.

To ensure the path-planning task is completed within a short timeframe, the maximum number of iterations *T* is set to 100, and the population size *N* is set to 50. The comparison in this section includes WOA variants alongside several mainstream optimization algorithms. The detailed results are presented below.

**Figure 8:**
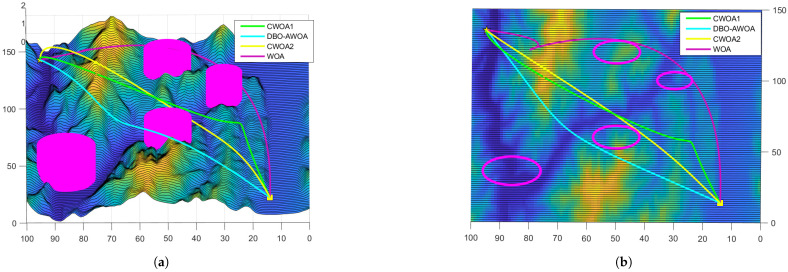
Comparison of simulation results 1. (**a**) Side view; (**b**) vertical view.

**Figure 9:**
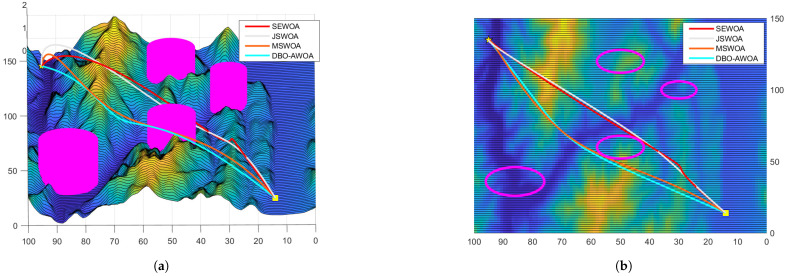
Comparison of simulation results 2. (**a**) Side view; (**b**) vertical view.

**Figure 10:**
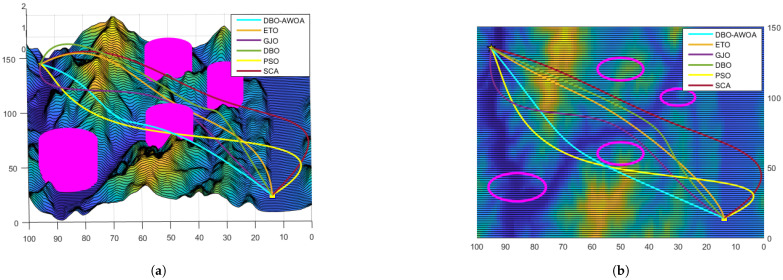
Comparison of simulation results 3. (**a**) Side view; (**b**) vertical view.

The simulation results presented in Figure 8 and Figure 9 confirm that all tested optimization algorithms are capable of generating feasible UAV paths. However, DBO-AWOA stands out by producing a smooth trajectory with a lower flight altitude, effectively minimizing energy consumption. In contrast, the standard WOA fails to generate a stable and safe route. Compared to other WOA variants, DBO-AWOA consistently delivers a safer, smoother, and more energy-efficient trajectory. As shown in Figure 10, DBO-AWOA generates a path characterized by shorter distance, moderate altitude, and superior smoothness. While ETO and DBO also produce viable paths, they exhibit significantly higher flight altitudes and steeper trajectories compared to DBO-AWOA.

**Figure 11:**
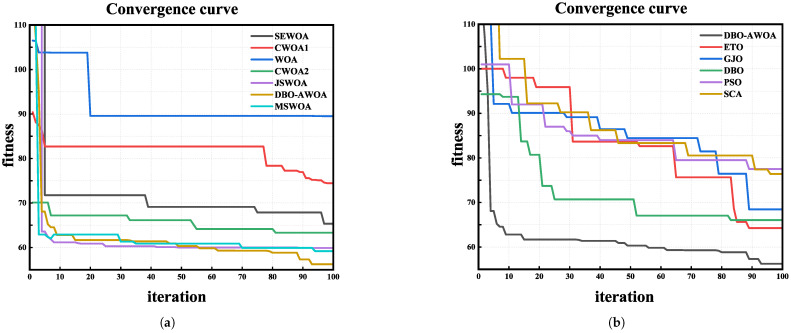
Comparison of fitness.

Figure 11a presents the fitness comparisons across the variants of WOA, further underscoring the superiority of DBO-AWOA in UAV path planning. The proposed approach effectively escapes local optima, demonstrating strong robustness. Among the tested methods, DBO-AWOA achieves the lowest fitness value of 56.22, outperforming MSWOA (59.19) and significantly surpassing WOA (89.51). Compared to other optimization strategies, DBO-AWOA improves fitness by 5.1% to 38.2%, highlighting its capability to generate optimal paths in complex mountainous terrains where other algorithms struggle or incur higher path costs.

Figure 11b presents the fitness comparisons across different algorithms. DBO-AWOA rapidly converges to the optimal path while efficiently refining the solution through local exploration. In contrast, other algorithms exhibit more erratic behavior, characterized by drastic fluctuations and higher randomness in optimization, leading to significantly less consistent path quality compared to DBO-AWOA.

To mitigate the influence of randomness in individual experiments and variations in weight selection, this study reports the average values and standard deviations of key performance indicators, ensuring a more robust evaluation. Each algorithm was executed 30 times, with the results summarized in Table 6.

DBO-AWOA exhibited superior performance across all evaluated indicators, achieving optimal mean values for path length (158.33), altitude threat (0.023), collision threat (2.124), and angle threat (2.313). Notably, it generated the shortest path among all algorithms. Its altitude threat was an order of magnitude lower than that of WOA (0.023 vs. 0.143) and significantly outperformed other methods, including SEWOA (4.053) and GJO (9.051), demonstrating its capability to maintain optimal flight altitudes while adhering to regulatory constraints.

The algorithm also demonstrated remarkable consistency, as reflected in its minimal standard deviations across key metrics (e.g., altitude threat: ±0.0039; angle threat: ±0.327). In contrast, methods such as CWOA2 and SEWOA exhibited substantial volatility in collision threat, suggesting unreliable obstacle avoidance in dynamic environments. DBO-AWOA achieved a collision threat of 2.124 ± 1.015, representing a 21.4% reduction compared to WOA (2.704 ± 0.583) and an 84.1% decrease relative to SEWOA (13.354 ± 6.927), further underscoring its robustness in trajectory optimization.

## 4. Conclusions

The DBO-AWOA algorithm effectively addresses the exploration–exploitation trade-off in three-dimensional UAV path planning. By incorporating chaotic initialization of the ICMIC, adaptive inertia weights, and reproductive mechanisms inspired by dung beetles, the algorithm demonstrates faster convergence rates and enhanced global search capabilities. Benchmark tests on the CEC2017 suite highlight its superiority, especially in unimodal and composition scenarios, where it outperforms traditional whale optimization variants and other algorithms. In 3D path-planning simulations, DBO-AWOA excels in generating energy-efficient, collision-free trajectories, with significant improvements in path smoothness and altitude adherence. Despite its strengths, challenges remain in hybrid functions with tightly coupled subcomponents, where excessive global exploration can slightly compromise precision. These results affirm the robustness of DBO-AWOA as a solution for complex optimization tasks while underscoring the importance of context-aware parameter adaptation to enhance stability in dynamic environments.

For practical deployment in a UAV system, the DBO-AWOA algorithm should be integrated with on-board sensors, such as LiDAR, GPS, and vision systems to provide real-time environmental awareness and dynamic obstacle avoidance. The UAV’s computational system must be able to process the algorithm’s complexity in real time, ensuring efficiency within the system’s operational constraints. Furthermore, the algorithm must be seamlessly coupled to the UAV flight control system, enabling continuous trajectory adjustments based on the latest sensor data.

## Figures and Tables

**Table 1:** The CEC2017 benchmark functions.

	No.	Functions
Unimodal Functions	1	Shifted and Rotated Bent Cigar Function
	3	Shifted and Rotated Zakharov Function
Simple Multimodal Functions	4	Shifted and Rotated Rosenbrock’s Function
	5	Shifted and Rotated Rastrigin’s Function
	6	Shifted and Rotated Schaffer’s F6 Function
	7	Shifted and Rotated Lunacek Bi-Rastrigin’s Function
	8	Shifted and Rotated Non-Continuous Rastrigin’s Function
	9	Shifted and Rotated Levy Function
	10	Shifted and Rotated Schwefel’s Function
Hybrid Functions	11	Hybrid Function 1(N = 3)
	12	Hybrid Function 2 (N = 3)
	13	Hybrid Function 3 (N = 3)
	14	Hybrid Function 4 (N = 4)
	15	Hybrid Function 5 (N = 4)
	16	Hybrid Function 6 (N = 4)
	17	Hybrid Function 7 (N = 5)
	18	Hybrid Function 8 (N = 5)
	19	Hybrid Function 9 (N = 5)
	20	Hybrid Function 10 (N = 6)
Composition Functions	21	Composition Function 1(N = 3)
	22	Composition Function 2 (N = 3)
	23	Composition Function 3 (N = 4)
	24	Composition Function 4 (N = 4)
	25	Composition Function 5 (N = 5)
	26	Composition Function 6 (N = 5)
	27	Composition Function 7 (N = 6)
	28	Composition Function 8 (N = 6)
	29	Composition Function 9 (N = 3)
	30	Composition Function 10 (N = 3)

**Table 2:** Parameter settings.

Algorithm	Parameter
WOA	d=30,T=500,N=30,b=1
JSWOA	$d=30,\;T=500,\;N=30,\;w_{\min}=0.04,\;w_{\max}=0.09$
SEWOA	d=30,T=500,N=30,k=2
MSWOA	d=30,T=500,N=30,k=0.3,n=1,CA=0.5
CWOA1	d=30,T=500,N=30,α=4
CWOA2	d=30,T=500,N=30,α=4

**Table 3:** Outcome of the benchmark function evaluation in F1–F14.

Functions	-	WOA	JSWOA	DBO-AWOA	SEWOA	MSWOA	CWOA1	CWOA2
F1	**Min**	$1.91\times 10^{9}$	$3.64\times 10^{10}$	$1.20\times 10^{9}$	$3.73\times 10^{9}$	$1.91\times 10^{10}$	$2.10\times 10^{9}$	$2.62\times 10^{9}$
	**Avg**	$4.70\times 10^{9}$	$4.06\times 10^{10}$	$3.44\times 10^{9}$	$9.88\times 10^{9}$	$2.69\times 10^{10}$	$4.92\times 10^{9}$	$5.69\times 10^{9}$
	**Std**	$3.38\times 10^{9}$	$4.73\times 10^{9}$	$2.90\times 10^{9}$	$2.13\times 10^{9}$	$3.83\times 10^{9}$	$3.15\times 10^{9}$	$4.61\times 10^{9}$
F3	**Min**	$1.02\times 10^{5}$	$7.39\times 10^{4}$	$4.31\times 10^{4}$	$1.45\times 10^{5}$	$8.20\times 10^{4}$	$1.44\times 10^{5}$	$1.50\times 10^{5}$
	**Avg**	$2.44\times 10^{5}$	$8.80\times 10^{4}$	$6.55\times 10^{4}$	$2.55\times 10^{5}$	$9.33\times 10^{4}$	$3.00\times 10^{5}$	$3.46\times 10^{5}$
	**Std**	$5.14\times 10^{4}$	$7.11\times 10^{3}$	$6.37\times 10^{3}$	$4.35\times 10^{4}$	$7.39\times 10^{4}$	$6.31\times 10^{4}$	$8.43\times 10^{4}$
F4	**Min**	$8.39\times 10^{2}$	$5.80\times 10^{3}$	$6.09\times 10^{2}$	$8.07\times 10^{2}$	$2.71\times 10^{3}$	$7.41\times 10^{2}$	$7.41\times 10^{2}$
	**Avg**	$1.36\times 10^{3}$	$7.21\times 10^{3}$	$1.30\times 10^{3}$	$1.83\times 10^{3}$	$5.58\times 10^{3}$	$1.32\times 10^{3}$	$1.32\times 10^{3}$
	**Std**	$1.22\times 10^{2}$	$6.00\times 10^{2}$	$7.82\times 10^{2}$	$8.64\times 10^{2}$	$2.07\times 10^{2}$	$1.74\times 10^{2}$	$1.74\times 10^{2}$
F5	**Min**	$7.75\times 10^{2}$	$8.56\times 10^{2}$	$7.40\times 10^{2}$	$7.63\times 10^{2}$	$8.46\times 10^{2}$	$7.90\times 10^{2}$	$7.53\times 10^{2}$
	**Avg**	$8.55\times 10^{2}$	$9.12\times 10^{2}$	$8.54\times 10^{2}$	$8.52\times 10^{2}$	$9.11\times 10^{2}$	$8.67\times 10^{2}$	$8.64\times 10^{2}$
	**Std**	$4.13\times 10^{1}$	$4.44\times 10^{1}$	$1.62\times 10^{1}$	$4.26\times 10^{1}$	$1.56\times 10^{1}$	$2.40\times 10^{1}$	$4.72\times 10^{1}$
F6	**Min**	$6.58\times 10^{2}$	$6.72\times 10^{2}$	$6.39\times 10^{2}$	$6.58\times 10^{2}$	$6.70\times 10^{2}$	$6.57\times 10^{2}$	$6.59\times 10^{2}$
	**Avg**	$6.76\times 10^{2}$	$6.91\times 10^{2}$	$6.57\times 10^{2}$	$6.82\times 10^{2}$	$6.89\times 10^{2}$	$6.81\times 10^{2}$	$6.81\times 10^{2}$
	**Std**	$1.11\times 10^{2}$	$6.74\times 10^{1}$	$1.05\times 10^{2}$	$1.66\times 10^{2}$	$8.89\times 10^{1}$	$7.53\times 10^{1}$	$1.17\times 10^{2}$
F7	**Min**	$1.16\times 10^{3}$	$1.34\times 10^{3}$	$1.15\times 10^{3}$	$1.20\times 10^{3}$	$1.28\times 10^{3}$	$1.19\times 10^{3}$	$1.16\times 10^{3}$
	**Avg**	$1.31\times 10^{3}$	$1.42\times 10^{3}$	$1.31\times 10^{3}$	$1.32\times 10^{3}$	$1.40\times 10^{3}$	$1.35\times 10^{3}$	$1.32\times 10^{3}$
	**Std**	$5.49\times 10^{1}$	$1.82\times 10^{1}$	$4.47\times 10^{1}$	$7.59\times 10^{1}$	$4.39\times 10^{1}$	$7.91\times 10^{1}$	$6.26\times 10^{1}$
F8	**Min**	$9.87\times 10^{2}$	$1.08\times 10^{3}$	$9.40\times 10^{2}$	$9.40\times 10^{2}$	$1.09\times 10^{3}$	$1.01\times 10^{3}$	$9.93\times 10^{2}$
	**Avg**	$1.07\times 10^{3}$	$1.12\times 10^{3}$	$1.06\times 10^{3}$	$1.06\times 10^{3}$	$1.15\times 10^{3}$	$1.08\times 10^{3}$	$1.08\times 10^{3}$
	**Std**	$2.78\times 10^{2}$	$5.66\times 10^{2}$	$6.06\times 10^{1}$	$3.26\times 10^{2}$	$7.82\times 10^{2}$	$3.02\times 10^{2}$	$2.13\times 10^{2}$
F9	**Min**	$6.38\times 10^{3}$	$8.11\times 10^{3}$	$5.81\times 10^{3}$	$7.07\times 10^{3}$	$6.24\times 10^{3}$	$6.62\times 10^{3}$	$6.97\times 10^{3}$
	**Avg**	$1.08\times 10^{4}$	$1.08\times 10^{4}$	$9.82\times 10^{3}$	$1.23\times 10^{4}$	$1.14\times 10^{4}$	$1.04\times 10^{4}$	$1.17\times 10^{4}$
	**Std**	$4.12\times 10^{3}$	$2.26\times 10^{3}$	$2.23\times 10^{3}$	$4.25\times 10^{3}$	$3.32\times 10^{6}$	$4.55\times 10^{3}$	$4.05\times 10^{3}$
F10	**Min**	$6.29\times 10^{3}$	$7.79\times 10^{3}$	$6.78\times 10^{3}$	$5.71\times 10^{3}$	$7.92\times 10^{3}$	$6.69\times 10^{3}$	$5.91\times 10^{3}$
	**Avg**	$7.63\times 10^{3}$	$8.64\times 10^{3}$	$8.22\times 10^{3}$	$7.33\times 10^{3}$	$8.88\times 10^{3}$	$7.64\times 10^{3}$	$7.87\times 10^{3}$
	**Std**	$1.89\times 10^{1}$	$2.47\times 10^{1}$	$1.37\times 10^{1}$	$1.53\times 10^{1}$	$1.59\times 10^{1}$	$1.12\times 10^{1}$	$1.17\times 10^{1}$
F11	**Min**	$3.77\times 10^{3}$	$6.23\times 10^{3}$	$2.82\times 10^{3}$	$2.82\times 10^{3}$	$4.16\times 10^{3}$	$3.39\times 10^{3}$	$2.94\times 10^{3}$
	**Avg**	$9.53\times 10^{3}$	$8.04\times 10^{3}$	$5.66\times 10^{3}$	$9.11\times 10^{3}$	$1.19\times 10^{4}$	$1.07\times 10^{4}$	$1.22\times 10^{4}$
	**Std**	$4.93\times 10^{3}$	$2.03\times 10^{3}$	$1.02\times 10^{3}$	$5.29\times 10^{3}$	$2.05\times 10^{3}$	$5.82\times 10^{3}$	$7.99\times 10^{3}$
F12	**Min**	$3.86\times 10^{7}$	$9.97\times 10^{9}$	$6.39\times 10^{8}$	$2.92\times 10^{7}$	$1.41\times 10^{9}$	$7.88\times 10^{7}$	$1.41\times 10^{8}$
	**Avg**	$4.92\times 10^{8}$	$1.12\times 10^{10}$	$1.71\times 10^{9}$	$7.10\times 10^{8}$	$3.54\times 10^{9}$	$4.69\times 10^{8}$	$5.03\times 10^{8}$
	**Std**	$6.56\times 10^{7}$	$2.04\times 10^{9}$	$2.70\times 10^{8}$	$5.25\times 10^{8}$	$2.11\times 10^{8}$	$2.10\times 10^{8}$	$3.83\times 10^{8}$
F13	**Min**	$2.13\times 10^{6}$	$2.48\times 10^{9}$	$4.14\times 10^{7}$	$2.04\times 10^{5}$	$6.84\times 10^{7}$	$4.15\times 10^{5}$	$6.97\times 10^{5}$
	**Avg**	$2.35\times 10^{7}$	$3.28\times 10^{9}$	$1.78\times 10^{8}$	$6.03\times 10^{7}$	$5.77\times 10^{8}$	$1.19\times 10^{7}$	$2.56\times 10^{7}$
	**Std**	$1.73\times 10^{7}$	$1.10\times 10^{9}$	$1.10\times 10^{8}$	$5.99\times 10^{7}$	$4.01\times 10^{8}$	$1.68\times 10^{7}$	$3.30\times 10^{7}$
F14	**Min**	$9.78\times 10^{4}$	$3.25\times 10^{6}$	$5.65\times 10^{4}$	$1.60\times 10^{5}$	$9.24\times 10^{4}$	$9.93\times 10^{4}$	$7.10\times 10^{4}$
	**Avg**	$1.90\times 10^{6}$	$1.61\times 10^{7}$	$1.72\times 10^{6}$	$3.35\times 10^{6}$	$3.41\times 10^{6}$	$2.32\times 10^{6}$	$3.14\times 10^{6}$
	**Std**	$3.62\times 10^{6}$	$2.29\times 10^{6}$	$3.28\times 10^{5}$	$1.26\times 10^{6}$	$2.97\times 10^{6}$	$9.43\times 10^{5}$	$1.39\times 10^{6}$
F15	**Min**	$2.91\times 10^{5}$	$5.85\times 10^{8}$	$2.19\times 10^{5}$	$2.43\times 10^{4}$	$1.73\times 10^{7}$	$1.76\times 10^{5}$	$6.82\times 10^{4}$
	**Avg**	$6.49\times 10^{6}$	$7.14\times 10^{8}$	$5.49\times 10^{6}$	$1.39\times 10^{7}$	$1.86\times 10^{8}$	$9.19\times 10^{6}$	$9.31\times 10^{6}$
	**Std**	$1.08\times 10^{6}$	$5.72\times 10^{6}$	$5.64\times 10^{6}$	$5.81\times 10^{6}$	$2.82\times 10^{6}$	$4.15\times 10^{6}$	$6.07\times 10^{6}$
F16	**Min**	$3.54\times 10^{3}$	$4.13\times 10^{3}$	$2.47\times 10^{3}$	$3.23\times 10^{3}$	$3.73\times 10^{3}$	$2.91\times 10^{3}$	$3.17\times 10^{3}$
	**Avg**	$4.44\times 10^{3}$	$5.51\times 10^{3}$	$4.29\times 10^{3}$	$4.32\times 10^{3}$	$5.01\times 10^{3}$	$4.33\times 10^{3}$	$4.59\times 10^{3}$
	**Std**	$2.63\times 10^{3}$	$1.37\times 10^{4}$	$2.58\times 10^{3}$	$2.93\times 10^{3}$	$3.72\times 10^{3}$	$4.75\times 10^{3}$	$6.81\times 10^{3}$
F17	**Min**	$2.04\times 10^{3}$	$3.84\times 10^{3}$	$2.04\times 10^{3}$	$2.09\times 10^{3}$	$2.35\times 10^{3}$	$2.05\times 10^{3}$	$2.21\times 10^{3}$
	**Avg**	$2.80\times 10^{3}$	$1.68\times 10^{4}$	$2.71\times 10^{3}$	$2.82\times 10^{3}$	$3.22\times 10^{3}$	$2.75\times 10^{3}$	$2.83\times 10^{3}$
	**Std**	$1.29\times 10^{3}$	$6.32\times 10^{3}$	$5.43\times 10^{2}$	$3.46\times 10^{3}$	$2.73\times 10^{3}$	$1.10\times 10^{3}$	$9.03\times 10^{2}$
F18	**Min**	$1.38\times 10^{6}$	$5.71\times 10^{5}$	$4.04\times 10^{5}$	$6.62\times 10^{5}$	$1.27\times 10^{6}$	$4.15\times 10^{5}$	$1.28\times 10^{6}$
	**Avg**	$1.75\times 10^{7}$	$4.85\times 10^{7}$	$1.20\times 10^{7}$	$1.76\times 10^{7}$	$4.69\times 10^{7}$	$2.32\times 10^{7}$	$2.84\times 10^{7}$
	**Std**	$3.06\times 10^{6}$	$8.78\times 10^{6}$	$2.17\times 10^{5}$	$3.09\times 10^{6}$	$1.71\times 10^{6}$	$5.27\times 10^{6}$	$5.99\times 10^{6}$
F19	**Min**	$2.74\times 10^{5}$	$1.44\times 10^{8}$	$7.73\times 10^{6}$	$7.17\times 10^{5}$	$3.94\times 10^{7}$	$2.12\times 10^{6}$	$8.26\times 10^{5}$
	**Avg**	$1.97\times 10^{7}$	$2.30\times 10^{8}$	$6.91\times 10^{7}$	$6.83\times 10^{7}$	$1.82\times 10^{8}$	$2.34\times 10^{7}$	$3.85\times 10^{7}$
	**Std**	$5.35\times 10^{6}$	$1.83\times 10^{7}$	$2.75\times 10^{6}$	$2.59\times 10^{6}$	$1.33\times 10^{6}$	$2.34\times 10^{6}$	$1.31\times 10^{6}$
F20	**Min**	$2.53\times 10^{3}$	$2.71\times 10^{3}$	$2.50\times 10^{3}$	$2.44\times 10^{3}$	$2.73\times 10^{3}$	$2.67\times 10^{3}$	$2.56\times 10^{3}$
	**Avg**	$2.94\times 10^{3}$	$3.03\times 10^{3}$	$2.92\times 10^{3}$	$2.98\times 10^{3}$	$3.15\times 10^{3}$	$2.95\times 10^{3}$	$3.04\times 10^{3}$
	**Std**	$5.32\times 10^{2}$	$3.43\times 10^{2}$	$3.61\times 10^{2}$	$5.97\times 10^{2}$	$2.61\times 10^{2}$	$3.27\times 10^{2}$	$4.08\times 10^{2}$
F21	**Min**	$2.57\times 10^{3}$	$2.64\times 10^{3}$	$2.51\times 10^{3}$	$2.51\times 10^{3}$	$2.65\times 10^{3}$	$2.53\times 10^{3}$	$2.52\times 10^{3}$
	**Avg**	$2.66\times 10^{3}$	$2.71\times 10^{3}$	$2.61\times 10^{3}$	$2.64\times 10^{3}$	$2.72\times 10^{3}$	$2.64\times 10^{3}$	$2.64\times 10^{3}$
	**Std**	$5.18\times 10^{2}$	$1.73\times 10^{2}$	$1.03\times 10^{2}$	$5.52\times 10^{2}$	$3.14\times 10^{2}$	$4.62\times 10^{2}$	$4.57\times 10^{2}$
F22	**Min**	$3.05\times 10^{3}$	$7.89\times 10^{3}$	$3.04\times 10^{3}$	$3.85\times 10^{3}$	$5.95\times 10^{3}$	$4.52\times 10^{3}$	$7.21\times 10^{3}$
	**Avg**	$8.21\times 10^{3}$	$9.39\times 10^{3}$	$8.05\times 10^{3}$	$8.06\times 10^{3}$	$9.26\times 10^{3}$	$8.67\times 10^{3}$	$9.01\times 10^{3}$
	**Std**	$3.05\times 10^{3}$	$6.80\times 10^{3}$	$2.40\times 10^{3}$	$2.28\times 10^{3}$	$5.84\times 10^{3}$	$1.63\times 10^{3}$	$4.77\times 10^{3}$
F23	**Min**	$2.92\times 10^{3}$	$3.18\times 10^{3}$	$2.88\times 10^{3}$	$2.91\times 10^{3}$	$3.13\times 10^{3}$	$3.01\times 10^{3}$	$3.01\times 10^{3}$
	**Avg**	$3.16\times 10^{3}$	$3.42\times 10^{3}$	$2.97\times 10^{3}$	$3.14\times 10^{3}$	$3.27\times 10^{3}$	$3.17\times 10^{3}$	$3.17\times 10^{3}$
	**Std**	$6.35\times 10^{2}$	$2.42\times 10^{2}$	$1.56\times 10^{2}$	$8.65\times 10^{2}$	$1.30\times 10^{2}$	$4.89\times 10^{2}$	$4.85\times 10^{2}$
F24	**Min**	$3.06\times 10^{3}$	$3.25\times 10^{3}$	$3.05\times 10^{3}$	$3.09\times 10^{3}$	$3.18\times 10^{3}$	$3.08\times 10^{3}$	$3.14\times 10^{3}$
	**Avg**	$3.26\times 10^{3}$	$3.71\times 10^{3}$	$3.12\times 10^{3}$	$3.30\times 10^{3}$	$3.43\times 10^{3}$	$3.27\times 10^{3}$	$3.27\times 10^{3}$
	**Std**	$3.31\times 10^{2}$	$3.95\times 10^{2}$	$1.32\times 10^{2}$	$1.56\times 10^{2}$	$1.56\times 10^{2}$	$9.26\times 10^{2}$	$5.97\times 10^{2}$
F25	**Min**	$3.12\times 10^{3}$	$3.75\times 10^{3}$	$3.08\times 10^{3}$	$3.08\times 10^{3}$	$3.46\times 10^{3}$	$3.09\times 10^{3}$	$3.12\times 10^{3}$
	**Avg**	$3.23\times 10^{3}$	$4.01\times 10^{3}$	$3.48\times 10^{3}$	$3.32\times 10^{3}$	$3.79\times 10^{3}$	$3.22\times 10^{3}$	$3.24\times 10^{3}$
	**Std**	$8.42\times 10^{2}$	$1.83\times 10^{3}$	$3.27\times 10^{2}$	$2.53\times 10^{2}$	$5.30\times 10^{2}$	$9.54\times 10^{2}$	$6.69\times 10^{2}$
F26	**Min**	$5.98\times 10^{3}$	$8.75\times 10^{3}$	$5.96\times 10^{3}$	$7.31\times 10^{3}$	$8.01\times 10^{3}$	$7.43\times 10^{3}$	$6.94\times 10^{3}$
	**Avg**	$8.55\times 10^{3}$	$9.97\times 10^{3}$	$7.29\times 10^{3}$	$8.72\times 10^{3}$	$9.45\times 10^{3}$	$8.72\times 10^{3}$	$8.77\times 10^{3}$
	**Std**	$3.48\times 10^{3}$	$1.58\times 10^{2}$	$5.83\times 10^{2}$	$2.22\times 10^{3}$	$2.65\times 10^{3}$	$1.57\times 10^{3}$	$1.22\times 10^{3}$
F27	**Min**	$3.33\times 10^{3}$	$3.20\times 10^{3}$	$3.20\times 10^{3}$	$3.33\times 10^{3}$	$3.41\times 10^{3}$	$3.33\times 10^{3}$	$3.31\times 10^{3}$
	**Avg**	$3.54\times 10^{3}$	$3.47\times 10^{3}$	$3.42\times 10^{3}$	$3.52\times 10^{3}$	$3.63\times 10^{3}$	$3.47\times 10^{3}$	$3.51\times 10^{3}$
	**Std**	$7.59\times 10^{2}$	$2.35\times 10^{2}$	$1.48\times 10^{2}$	$1.79\times 10^{2}$	$2.23\times 10^{2}$	$5.23\times 10^{2}$	$2.16\times 10^{2}$
F28	**Min**	$3.53\times 10^{3}$	$3.33\times 10^{3}$	$3.30\times 10^{3}$	$3.44\times 10^{3}$	$4.04\times 10^{3}$	$3.50\times 10^{3}$	$3.43\times 10^{3}$
	**Avg**	$4.09\times 10^{3}$	$5.35\times 10^{3}$	$4.91\times 10^{3}$	$4.10\times 10^{3}$	$5.12\times 10^{3}$	$4.04\times 10^{3}$	$4.30\times 10^{3}$
	**Std**	$9.61\times 10^{2}$	$1.16\times 10^{3}$	$1.77\times 10^{3}$	$1.30\times 10^{3}$	$2.42\times 10^{3}$	$1.79\times 10^{3}$	$1.17\times 10^{3}$
F29	**Min**	$4.31\times 10^{3}$	$4.97\times 10^{3}$	$4.02\times 10^{3}$	$4.05\times 10^{3}$	$5.27\times 10^{3}$	$4.59\times 10^{3}$	$4.60\times 10^{3}$
	**Avg**	$5.27\times 10^{3}$	$6.21\times 10^{3}$	$5.02\times 10^{3}$	$5.73\times 10^{3}$	$6.17\times 10^{3}$	$5.38\times 10^{3}$	$5.34\times 10^{3}$
	**Std**	$1.71\times 10^{3}$	$2.41\times 10^{3}$	$5.58\times 10^{2}$	$1.30\times 10^{3}$	$3.31\times 10^{3}$	$3.68\times 10^{3}$	$2.48\times 10^{3}$
F30	**Min**	$6.33\times 10^{6}$	$1.55\times 10^{9}$	$1.67\times 10^{7}$	$1.58\times 10^{6}$	$9.25\times 10^{7}$	$6.98\times 10^{6}$	$2.18\times 10^{7}$
	**Avg**	$8.19\times 10^{7}$	$3.41\times 10^{9}$	$1.30\times 10^{8}$	$5.79\times 10^{7}$	$2.75\times 10^{8}$	$7.19\times 10^{7}$	$9.28\times 10^{7}$
	**Std**	$2.78\times 10^{7}$	$6.62\times 10^{9}$	$8.64\times 10^{7}$	$3.47\times 10^{7}$	$1.72\times 10^{7}$	$2.53\times 10^{7}$	$5.90\times 10^{7}$

**Table 4:** Performance comparison between different algorithms.

Functions	-	DBO-AWOA	ETO	GJO	DBO	PSO	SCA
F3	**Min**	$4.31\times 10^{4}$	$4.68\times 10^{4}$	$4.91\times 10^{4}$	$6.04\times 10^{4}$	$4.69\times 10^{4}$	$5.08\times 10^{4}$
	**Avg**	$6.55\times 10^{4}$	$6.43\times 10^{4}$	$6.48\times 10^{4}$	$8.46\times 10^{4}$	$8.12\times 10^{4}$	$8.64\times 10^{4}$
	**Std**	$6.37\times 10^{3}$	$1.22\times 10^{4}$	$1.11\times 10^{4}$	$9.89\times 10^{3}$	$2.48\times 10^{4}$	$2.17\times 10^{4}$
F5	**Min**	$7.40\times 10^{2}$	$6.94\times 10^{2}$	$6.63\times 10^{2}$	$5.99\times 10^{2}$	$7.02\times 10^{2}$	$7.12\times 10^{2}$
	**Avg**	$8.54\times 10^{2}$	$8.76\times 10^{2}$	$8.77\times 10^{2}$	$8.60\times 10^{2}$	$9.42\times 10^{2}$	$9.20\times 10^{2}$
	**Std**	$1.62\times 10^{1}$	$4.95\times 10^{1}$	$4.17\times 10^{1}$	$7.44\times 10^{1}$	$9.68\times 10^{1}$	$7.20\times 10^{1}$
F7	**Min**	$1.15\times 10^{3}$	$1.22\times 10^{3}$	$1.20\times 10^{3}$	$1.04\times 10^{3}$	$1.04\times 10^{3}$	$1.18\times 10^{3}$
	**Avg**	$1.31\times 10^{3}$	$1.32\times 10^{3}$	$1.48\times 10^{3}$	$1.35\times 10^{3}$	$1.47\times 10^{3}$	$1.53\times 10^{3}$
	**Std**	$4.47\times 10^{1}$	$6.68\times 10^{1}$	$6.26\times 10^{1}$	$7.87\times 10^{1}$	$7.68\times 10^{1}$	$9.05\times 10^{1}$
F12	**Min**	$6.39\times 10^{8}$	$9.27\times 10^{7}$	$8.57\times 10^{7}$	$1.33\times 10^{6}$	$2.55\times 10^{8}$	$1.04\times 10^{9}$
	**Avg**	$1.71\times 10^{9}$	$5.66\times 10^{8}$	$9.98\times 10^{8}$	$5.52\times 10^{7}$	$4.27\times 10^{8}$	$2.61\times 10^{9}$
	**Std**	$2.70\times 10^{8}$	$5.12\times 10^{8}$	$9.39\times 10^{8}$	$1.08\times 10^{8}$	$2.23\times 10^{8}$	$8.64\times 10^{8}$
F13	**Min**	$4.14\times 10^{7}$	$1.37\times 10^{7}$	$2.88\times 10^{5}$	$4.74\times 10^{4}$	$8.15\times 10^{7}$	$3.72\times 10^{8}$
	**Avg**	$1.78\times 10^{8}$	$1.62\times 10^{8}$	$3.05\times 10^{8}$	$2.08\times 10^{8}$	$1.81\times 10^{8}$	$1.10\times 10^{9}$
	**Std**	$1.10\times 10^{8}$	$4.76\times 10^{8}$	$3.06\times 10^{8}$	$2.01\times 10^{8}$	$6.29\times 10^{8}$	$4.11\times 10^{8}$
F22	**Min**	$3.04\times 10^{3}$	$3.12\times 10^{3}$	$3.28\times 10^{3}$	$3.36\times 10^{3}$	$5.77\times 10^{3}$	$3.85\times 10^{3}$
	**Avg**	$8.05\times 10^{3}$	$8.19\times 10^{3}$	$9.17\times 10^{3}$	$8.93\times 10^{3}$	$1.08\times 10^{4}$	$9.39\times 10^{3}$
	**Std**	$2.40\times 10^{3}$	$2.48\times 10^{3}$	$2.50\times 10^{3}$	$4.34\times 10^{3}$	$5.21\times 10^{3}$	$4.29\times 10^{3}$
F26	**Min**	$5.96\times 10^{3}$	$6.84\times 10^{3}$	$7.78\times 10^{3}$	$7.71\times 10^{3}$	$8.85\times 10^{3}$	$6.62\times 10^{3}$
	**Avg**	$7.29\times 10^{3}$	$6.91\times 10^{3}$	$6.07\times 10^{3}$	$7.01\times 10^{3}$	$9.47\times 10^{3}$	$7.70\times 10^{3}$
	**Std**	$5.83\times 10^{2}$	$6.83\times 10^{2}$	$7.66\times 10^{2}$	$6.28\times 10^{2}$	$9.31\times 10^{2}$	$4.85\times 10^{2}$
F29	**Min**	$4.02\times 10^{3}$	$4.15\times 10^{3}$	$4.81\times 10^{3}$	$4.32\times 10^{3}$	$4.15\times 10^{3}$	$4.45\times 10^{3}$
	**Avg**	$5.02\times 10^{3}$	$5.59\times 10^{3}$	$6.30\times 10^{3}$	$5.36\times 10^{3}$	$5.42\times 10^{3}$	$5.12\times 10^{3}$
	**Std**	$5.58\times 10^{2}$	$6.30\times 10^{2}$	$7.11\times 10^{2}$	$8.21\times 10^{2}$	$9.42\times 10^{2}$	$7.50\times 10^{2}$

**Table 5:** Ablation experiments of DBO-AWOA.

Functions	-	DBO-AWOA	WOA	IWOA	NWOA	AWOA	DWOA
F3	Min	$4.31\times 10^{4}$	$1.02\times 10^{5}$	$2.18\times 10^{5}$	$9.64\times 10^{4}$	$7.57\times 10^{4}$	$5.74\times 10^{4}$
	Avg	$6.55\times 10^{4}$	$2.44\times 10^{5}$	$3.98\times 10^{5}$	$2.75\times 10^{5}$	$8.92\times 10^{4}$	$8.92\times 10^{4}$
	Std	$6.37\times 10^{3}$	$5.14\times 10^{4}$	$8.28\times 10^{4}$	$8.39\times 10^{4}$	$5.12\times 10^{3}$	$8.88\times 10^{3}$
F5	Min	$7.40\times 10^{2}$	$7.75\times 10^{2}$	$7.76\times 10^{2}$	$7.57\times 10^{2}$	$8.03\times 10^{2}$	$7.66\times 10^{2}$
	Avg	$8.54\times 10^{2}$	$8.55\times 10^{2}$	$8.55\times 10^{2}$	$8.63\times 10^{2}$	$8.77\times 10^{2}$	$8.57\times 10^{2}$
	Std	$1.62\times 10^{1}$	$4.13\times 10^{1}$	$5.56\times 10^{1}$	$7.55\times 10^{1}$	$4.51\times 10^{1}$	$4.22\times 10^{1}$
F7	Min	$1.15\times 10^{3}$	$1.16\times 10^{3}$	$1.20\times 10^{3}$	$1.15\times 10^{3}$	$1.22\times 10^{3}$	$1.19\times 10^{3}$
	Avg	$1.31\times 10^{3}$	$1.31\times 10^{3}$	$1.33\times 10^{3}$	$1.33\times 10^{3}$	$1.33\times 10^{3}$	$1.31\times 10^{3}$
	Std	$4.47\times 10^{1}$	$5.49\times 10^{1}$	$8.95\times 10^{1}$	$8.18\times 10^{1}$	$7.06\times 10^{1}$	$5.76\times 10^{1}$
F12	Min	$6.39\times 10^{8}$	$3.86\times 10^{7}$	$1.09\times 10^{8}$	$9.61\times 10^{7}$	$4.76\times 10^{8}$	$8.23\times 10^{8}$
	Avg	$1.71\times 10^{9}$	$4.92\times 10^{8}$	$5.86\times 10^{8}$	$4.16\times 10^{8}$	$1.34\times 10^{9}$	$1.69\times 10^{9}$
	Std	$2.70\times 10^{8}$	$6.56\times 10^{7}$	$3.99\times 10^{8}$	$3.10\times 10^{8}$	$5.89\times 10^{8}$	$5.63\times 10^{8}$
F13	Min	$4.14\times 10^{7}$	$2.13\times 10^{6}$	$7.63\times 10^{5}$	$4.12\times 10^{5}$	$1.27\times 10^{7}$	$1.56\times 10^{7}$
	Avg	$1.78\times 10^{8}$	$2.35\times 10^{7}$	$1.09\times 10^{7}$	$1.01\times 10^{7}$	$1.38\times 10^{8}$	$1.20\times 10^{8}$
	Std	$1.10\times 10^{8}$	$2.73\times 10^{7}$	$1.05\times 10^{7}$	$1.39\times 10^{7}$	$1.07\times 10^{8}$	$6.29\times 10^{7}$
F22	Min	$3.04\times 10^{3}$	$3.05\times 10^{3}$	$3.31\times 10^{3}$	$3.95\times 10^{3}$	$4.53\times 10^{3}$	$3.52\times 10^{3}$
	Avg	$8.05\times 10^{3}$	$8.21\times 10^{3}$	$8.58\times 10^{3}$	$8.17\times 10^{3}$	$8.98\times 10^{3}$	$8.45\times 10^{3}$
	Std	$2.40\times 10^{3}$	$3.05\times 10^{3}$	$1.47\times 10^{3}$	$2.60\times 10^{3}$	$1.60\times 10^{3}$	$2.43\times 10^{3}$
F26	Min	$5.96\times 10^{3}$	$5.98\times 10^{3}$	$6.91\times 10^{3}$	$6.12\times 10^{3}$	$6.95\times 10^{3}$	$6.54\times 10^{3}$
	Avg	$7.29\times 10^{3}$	$8.55\times 10^{3}$	$8.82\times 10^{3}$	$8.46\times 10^{3}$	$8.84\times 10^{3}$	$7.38\times 10^{3}$
	Std	$5.83\times 10^{2}$	$3.48\times 10^{3}$	$1.12\times 10^{3}$	$1.19\times 10^{3}$	$8.39\times 10^{2}$	$8.61\times 10^{2}$
F29	Min	$4.02\times 10^{3}$	$4.31\times 10^{3}$	$4.73\times 10^{3}$	$4.16\times 10^{3}$	$4.66\times 10^{3}$	$4.51\times 10^{3}$
	Avg	$5.02\times 10^{3}$	$5.27\times 10^{3}$	$5.69\times 10^{3}$	$5.29\times 10^{3}$	$5.68\times 10^{3}$	$5.24\times 10^{3}$
	Std	$5.58\times 10^{2}$	$1.71\times 10^{3}$	$6.03\times 10^{2}$	$5.93\times 10^{2}$	$5.92\times 10^{2}$	$6.66\times 10^{2}$

**Table 6:** Comparison of different indicators.

Algorithm	-	*Length*	*Altitude Threat*	*Collision Threat*	*Angle Threat*
DBO-AWOA	Mean	158.33	0.023	2.124	2.313
	Std	7.313	0.0039	1.015	0.327
WOA	Mean	186.917	0.143	2.704	4.231
	Std	11.684	0.051	0.583	1.234
CWOA1	Mean	171.103	1.025	4.314	0.312
	Std	11.313	0.374	0.951	0.111
CWOA2	Mean	168.342	1.557	10.429	5.319
	Std	3.809	0.0323	5.132	1.136
SEWOA	Mean	172.771	4.053	13.354	11.642
	Std	8.123	1.614	6.927	3.423
JSWOA	Mean	160.348	0.317	3.165	3.115
	Std	6.611	0.373	1.612	1.031
MSWOA	Mean	164.933	5.114	3.178	3.101
	Std	5.366	1.129	1.1322	0.570
ETO	Mean	161.101	7.231	1.824	2.364
	Std	8.316	2.632	0.773	1.099
GJO	Mean	171.259	9.051	9.997	6.444
	Std	11.399	5.329	5.315	2.222
DBO	Mean	166.149	8.225	1.344	3.032
	Std	3.674	2.513	0.333	1.331
PSO	Mean	176.339	6.337	1.714	5.131
	Std	5.521	2.433	0.623	1.151
SCA	Mean	180.331	9.526	4.215	7.131
	Std	8.339	3.161	1.193	4.311

## Data Availability

The data used in this study are not public but available upon request.
